# pH responsive curcumin released from urea-amine group functionalized mesoporous organosilica nanoparticles

**DOI:** 10.55730/1300-0527.3632

**Published:** 2023-08-13

**Authors:** Osman Tayyar ARLI, Yaşar GÖK, Halil Zeki GÖK

**Affiliations:** Department of Chemistry, Faculty of Science and Letters, Burdur Mehmet Akif Ersoy University, Burdur, Turkiye

**Keywords:** Mesoporous material, MCM-41, MON, curcumin, drug loading and release

## Abstract

Studies on natural products with anticancer properties have gained more importance in recent years in order to not damage healthy tissues during cancer treatments or to perform the treatment causing the least damage. Curcumin is a common natural product with anticancer properties, but its low solubility, instability, and bioavailability have limited its use in clinical applications in cancer research. As a proposed solution to this problem, a new mesoporous organosilica nanocarrier (**MON-A**) functionalized with a 1,2-diphenylethane-1,2-diamine structure capable of pH-controlled release was prepared in this study. **MON-A** was characterized by TGA, BET, XRD, FT-IR, and SEM-EDS analyses. Dispersion of **MON-A** into curcumin stock solution in ethanol afforded a curcumin-loaded **MON-A-Cur** system. Elevated loading capacity and pH-controlled release were provided by the Schiff base reaction that occurred during loading of curcumin with 1,2-diphenylethane-1,2-diamine placed on the silica wall of the nanocarrier system. The encapsulation efficiency was 25% for **MON-A-Cur**. In in vitro release experiments, curcumin release from the **MON-A-Cur** system was 0.5% at physiological and endosomal pH values. The resulting low release percentage indicates the presence of very strong interactions between the nanocarrier **MON-A** and curcumin. This strong interaction showed that **MON-A** nanocarrier could carry 99.5% of the curcumin without leakage under physiological and endosomal pH conditions without using pore capping agents. At a lower acidic pH value (pH 4.5), 26.3% curcumin release was obtained. These findings showed that the cumulative release of curcumin from the **MON-A-Cur** system can be achieved in a long-term and pH-controlled manner.

## 1. Introduction

Curcumin is a compound found in turmeric, a commonly used spice in cooking. It has been studied for its potential anticancer properties and has been found to inhibit the growth of certain types of cancer cells in laboratory studies [1–3]. It may also have antiinflammatory [4] and antioxidant effects [5]. However, despite all these potential properties of curcumin, the stability and bioavailability of curcumin were found to be quite weak in Phase-I/Phase-II clinical studies in humans [6]. This constitutes a serious problem that limits the use of curcumin in treatment. The level of curcumin in plasma and tissue remains low due to factors such as low absorption caused by its low water solubility, quick metabolism, instability at physiological pH, and fast systemic elimination [7]. In addition to providing stability of curcumin with chemical modifications [6], various nanocarrier systems have come into use to increase the therapeutic effect of curcumin. Among the nanocarrier systems, curcumin-loaded mesoporous materials have been studied in several forms, including silica, titanium dioxide, and iron oxide [8–10]. These materials have been found to increase the solubility, stability, and bioavailability of curcumin, which can improve its efficacy as an anticancer agent [11–13]. Additionally, the mesoporous structure of these materials can be utilized for targeted drug delivery [14], which can help to reduce the toxic effects on normal cells and increase the efficacy of the treatment.

Mesoporous silica drug carriers with pore sizes ranging from 2 to 50 nm emerge as an important material among mesoporous materials because they allow properties such as pore volume and diameter, surface area, and hydrophobicity to be adjusted as desired [15]. These are important parameters that directly affect drug loading and release studies [16]. Among these parameters, the type of interaction between the drug molecule and the silica surface is important for drug loading and release studies. Drugs interact with silica surfaces by electrostatic or hydrogen bond formation mechanisms, depending on the nature of the drug and the pH of the environment [17]. Strong coactions between the drug and silica occur with elevated drug loading and prolonged drug release profiles. Conversely, weak drug–silica interactions occur with low drug loading and immediate release profiles. Hydrogen bonds between silanol and drug are very low in strength and are not sufficient to obtain sustained drug release. Therefore, by functionalizing the silanol groups on the silica surface with different organic silanes, the hydrophobicity of the silica material is increased and, in this way, new silica species are synthesized that can create stronger interactions with the drug molecule [18,19].

Due to the advantages of mesoporous silica materials listed above, the number of studies on curcumin-loaded mesoporous silica materials has started to increase [20–23]. Some of these studies were carried out to reveal the effects of functionalization of the silica surface with organic structures containing amino groups on curcumin loading and release [24–26]. For example, Atiyah et al. synthesized MCM-41 [24] and SBA-15 [25] and then functionalized them with 3-aminopropyltriethoxysilane (APTES) to obtain mesoporous materials NH_2_@MCM-41 and NH_2_@SBA-15. Then they loaded curcumin into these materials with the help of noncovalent interactions. In both studies, it was revealed that more curcumin loading can be made on materials whose surfaces are functionalized with APTES. In subsequent drug release studies, it was determined that the release of curcumin from NH_2_@MCM-41 and NH_2_@SBA-15 materials took place in a controlled manner over a longer time due to increased intermolecular interactions between APTES and curcumin. In addition, whether the number of amino groups has an effect on curcumin loading and release was investigated in a study by a different group [26]. It was determined that the curcumin loading rate increased from 3.6% to 33.5% when an organic group containing two amino groups was used instead of an organic group containing a single amino group during the functionalization of MSN. These studies have revealed that functionalization of the carrier material with an organic group containing an amino group is a rational approach in order to achieve effective transport and controlled release of curcumin. Although studies on the functionalization of materials such as MCM-41 and SBA-15 with organic groups containing amino groups are reported in the literature, the number of studies on the functionalization of mesoporous organosilica materials with amino groups and their use in curcumin transport is quite limited.

In the present study, we report a long-term and pH-controlled curcumin loaded mesoporous organosilica drug nanocarrier system (**MON-A-Cur)**. The mesoporous nanocarrier was functionalized with monosilyl derivative of 1,2-diphenylethane-1,2-diamine first. This group has the potential to form acid-labile imine bonds with the keto group in curcumin, as well as having groups that can cause many types of intermolecular interactions with curcumin. Then, to obtain a curcumin-loaded mesoporous organosilica nanocarrier system (**MON-A-Cur**), curcumin was loaded to **MON-A** by stirring curcumin and **MON-A** in the dark for 24 h. Additionally, pH-controlled release of curcumin-loaded mesoporous organosilica materials (**MON-A-Cur**) was evaluated by in vitro experiments at different pHs. Overall, the present study describes an alternative approach to increase the bioavailability of hydrophobic natural anticancer products such as curcumin and achieve pH-controlled release.

It is important to note that curcumin-loaded mesoporous materials and pH-controlled release are still in the preclinical research stage and more studies are needed to investigate their effectiveness and safety as a cancer therapy before use in clinical applications.

## 2. Experimental

### 2.1. Materials

The urea-amine ligand **1** and reference material **MCM-41** were synthesized by following the method given in the literature [27,28]. Cetyltrimethylammonium bromide (CTAB), tetraethylortosilicate (TEOS), curcumin, and all the other materials were purchased from commercial suppliers (Merck or Sigma-Aldrich). All the materials used in the experiments were analytically pure. Drying and refinement of the solvents used were performed as described by Perrin and Armarego [29].

### 2.2. Equipment for measurements

FTIR was performed using a Shimadzu IR Affinity 1S spectrometer with the KBr pellet technique. A PANalytical Empyrean X-ray diffraction meter using Cu-Kα radiation (λ = 0.154056 nm) in the range of 1°–10° and 10°–80° scattering angle 2θ was used for X-ray diffraction (XRD) measurements. The field emission scanning electron microscope images of the samples were obtained using a JEOL JEM1400 PLUS device. Thermogravimetric analysis measurements were performed on a Seiko SII TG/DTA 7200 instrument. The flow rate of N_2_ gas was 2 mL min^−1^ and the heat rate was 10 °C min^−1^ during the TGA analysis. N_2_ sorption isotherms were measured on a Micrometrics Surface Area and Porosity Tristar II analyzer. The Brunauer–Emmett–Teller method was used to calculate the surface area of the mesoporous materials, and the Barrett, Joyner, and Halenda method was used to determine the pore size distribution. The UV-Vis measurements in drug loading and release experiments were performed on an Optima 3000 UV-Vis spectrophotometer.

### 2.3. Preparation of MCM-41 and MON-A

#### 2.3.1. Preparation of MCM-41

CTAB (3 g, 8.23 mmol) and ultrapure water (1440 mL) were placed in a three-necked flask and mixed at 41 °C until a neat solution was formed. After adding 10.5 mL of 2 M NaOH solution to the flask, the temperature was brought to 80 °C. The reaction mixture was stirred at this temperature for 1 h. At the end of this period, 15 mL of tetraethyl orthosilicate (TEOS) in a dropping funnel was added to the reaction mixture dropwise. After addition of TEOS, the reaction mixture continued to be stirred at 80 °C for another 3 h. At the end of this time, the heating of the mixer was turned off and the reaction continued to be stirred. The reaction temperature was allowed to reach room temperature. Then the reaction mixture was filtered through a crucible. After the remaining white solid was cleansed with distilled water and ethanol, it was dehydrated in an oven at 55 °C for 48 h. The calcination of the oven-dried solid part at 550 °C for 6 h yielded 3 g of the final mesoporous MCM-41 as a white solid.

#### 2.3.2. Preparation of MON-A

**MON-A** was synthesized following the method published by our group [30]. The general procedure of the sol–gel method was used in this. First, ultrapure water was transferred to a three-necked flask and then CTAB (0.2 g, 0.53 mmol) was added. The temperature of the reaction mixture was increased to 40 °C and mixing was started. After 2 M NaOH (0.7 mL) was added to the reaction mixture, the temperature was adjusted to 80 °C. Next, compound **1** (0.23 g, 0.5 mmol) was dissolved in 1 mL of ethanol and it was started to be dropped into the mixture with TEOS (1.04 g, 5 mmol) through separate syringes over 20 min. After the drips were finished, the mixture was left to mix at 80 °C for 3 h. After 3 h, mixing was stopped and the reaction mixture was left to mature for 2 days at 80 °C. The solid part in the reaction mixture was filtered, cleansed with distilled water, and placed to dry under vacuum in an oven at 60 °C; 200 mL of ethanol and 3.4 mL of HCl were added per 1 g of mesoporous material followed by mixing for 7 h at 60 °C. After 7 h, the solid part was filtered from the mixture and cleansed with distilled water and ethanol. The resulting solid part was then dried in an oven at 60 °C. Finally, this fraction obtained was mixed with 0.01 M NaOH (300 mL) for 30 min. It was filtered again, cleansed, and dried under vacuum, yielding 0.35 g of mesoporous organosilica nanoparticles.

### 2.4. Loading and release of curcumin

#### 2.4.1. Preparation of curcumin-loaded materials

The methods in the literature were applied to load the natural anticancer drug curcumin into the prepared **MON-A** and **MCM-41** structures [31]. According to the method, a stock solution with a concentration of 4 mg curcumin/mL ethanol was prepared. Then 100 mg of **MON-A** or **MCM-41** was added to the 25 mL of curcumin solution and dispersed using a sonicator. The solutions obtained after dispersion were mixed at room temperature for 24 h in darkness. After this period, the mixture was centrifuged to separate the drug loading solution. The remaining drug loaded mesoporous material (hereinafter called **MON-A-Cur** and **Cur@MCM-41**) was washed with 20 mL of ethanol and centrifuged. MON-A-Cur and Cur@MCM-41 were then dried under vacuum. The amount of loaded drug and loading capacity were calculated using equations (1) and (2) after reading the absorbance of the loading and washing solutions in the UV-Vis spectrophotometer at λ = 420 nm [16,32]. In order to calculate the amount of curcumin corresponding to the absorption values read in the spectrophotometer, a previously prepared standard calibration curve of curcumin was used.


(1) $$Encapsulation\;Efficiency\;(\%)=\frac{the\;amount\;of\;drug\;loaded\;(mg)}{the\;intial\;amount\;of\;drug\;(mg)}\times 100$$

(2) $$Loading\;capacity\;(\%)=\frac{the\;amount\;of\;drug\;loaded\;(mg)}{the\;amount\;of\;drug\;loaded\;(mg)+the\;amount\;of\;MON\;(mg)}\times 100$$

#### 2.4.2. Curcumin release

For curcumin release studies, 1 mg of **MON-A-Cur** was dispersed in 10 mL of pH 7.4 or pH 5.5 solutions containing 10% (v/v) Tween-80. The resulting mixture was stirred regularly at 100 rpm and 37 °C. While mixing was in progress, samples were taken from the drug release medium at regular intervals to determine the amount of drug released. For this, the sample was first centrifuged at 12,000 rpm for 15 min. The UV-Vis readings of the supernatant portion were then read at λ = 420 nm. After reading, the supernatant was returned to the release medium. The amount of drug released was calculated with the help of a standard calibration curve. The same method given above was followed for the curcumin release from **Cur@MCM-41**.

## 3. Results and discussion

### 3.1. Characterization

**Figure 1:**
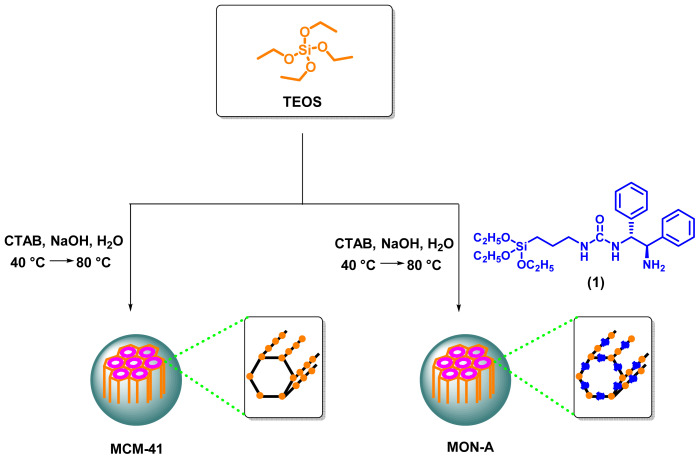
Synthetic routes of mesoporous materials **MCM-41** and **MON-A**.

The mesoporous structures **MCM-41** and **MON-A** were synthesized according to the route shown in Figure 1. Although various methods for the synthesis of **MCM-41** have been published in the literature, the method reported by Nikoorazm et al. was followed in the present study [28]. Mesoporous organosilica material **MON-A** was synthesized using TEOS and monosilyl compound **1** as silicon sources at a mole ratio of 10:1, following the same method used in the synthesis of **MCM-41**. The synthesized mesoporous materials **MCM-41** and **MON-A** were structurally and morphologically characterized with FTIR, FE-SEM, TGA, BET, and XRD analyses.

**Figure 2:**
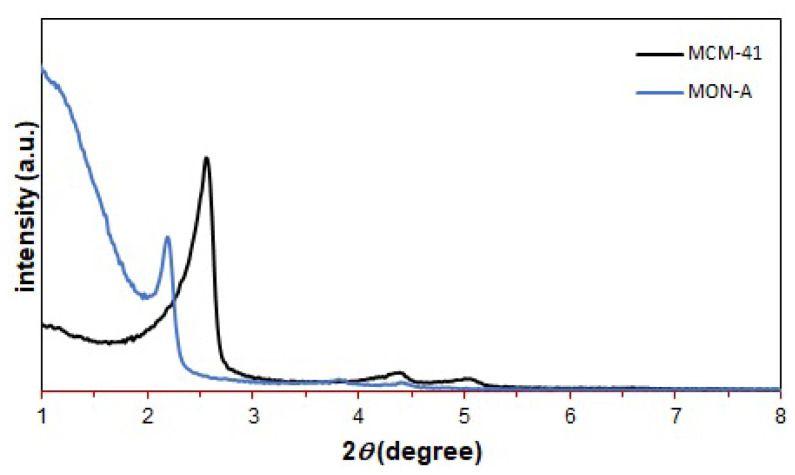
Low angle X-ray diffraction (XRD) patterns of **MCM-41** and **MON-A**.

Firstly, XRD measurement for the synthesized mesoporous materials was performed. The low angle powder X-ray diffraction pattern for **MCM-41** and **MON-A** is shown in Figure 2. The appearance of a strong signal at 2θ = 2.6° and two lower intensity signals at 2θ = 4.4° and 5.0° in the low angle XRD powder pattern of **MCM-41** indicates a hexagonal arrangement for it [18,33]. The XRD powder pattern structure recorded for **MON-A** was quite similar to that obtained for **MCM-41**. However, the reflection signals for **MON-A** shifted to smaller angles and were observed with lower intensity at 2θ = 2.2°, 3.8°, and 4.4°. These results show that compound **1** was successfully adjoined to the silica network structure of **MON-A**.

**Figure 3:**
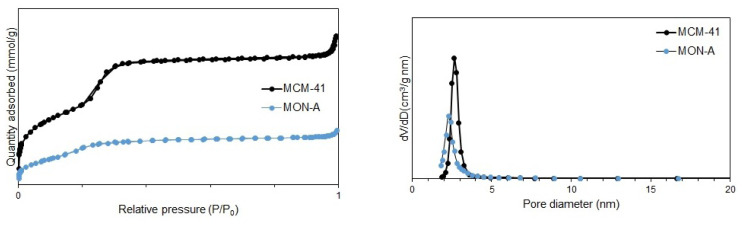
N_2_ adsorption–desorption isotherms (on left) and pore size distributions (on right) of **MCM-41** and **MON-A**.

The absorption–desorption isotherms and pore size distribution curves for **MCM-41** and **MON-A** are given in Figure 3. When the absorption–desorption isotherms obtained for **MCM-41** and **MON-A** are examined, they correspond to the type IV isotherm with an H1 hysteresis loop according to the IUPAC classification [34]. The Table shows the total pore volume (V_total_), Brunauer–Emmett–Teller surface area (S_BET_), and pore diameter (D_BJH_) values for **MCM-41** and **MON-A**. Decreases in the pore size, pore volume, and total surface area values for **MON-A** were observed compared with the values of the bare **MCM-41**. This result confirms that the cocondensation reaction successfully occurred and **MON-A** was formed.

**Figure 4:**
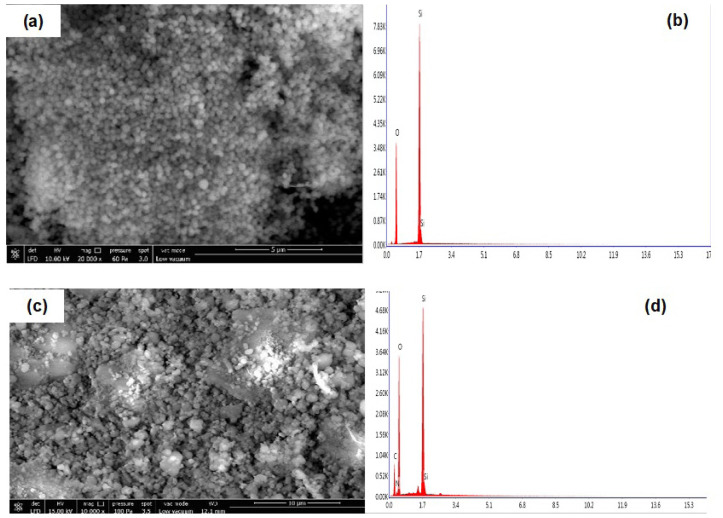
Field emission scanning electron microscope (FE-SEM) images of **MCM-41** (a) and **MON-A** (c); and EDS analysis of **MCM-41** (b) and **MON-A** (d).

Field emission scanning electron microscopy and energy dispersive spectroscopy were performed to determine the morphology of **MCM-41** and **MON-A**. The FE-SEM images and EDS spectra of **MCM-41** and **MON-A** are given in Figure 4. The FE-SEM analysis showed that MCM-41 has a surface morphology in the form of nanospheres and EDS analysis showed that only Si and O atoms are present in its structure. When the FE-SEM image of **MON-A** was examined, it was observed that **MON-A** formed agglomerations and, as a result, it had a more irregular surface morphology with different sizes rather than a uniform regular nanosphere shape like **MCM-41**. The EDS spectrum of **MON-A** shows that the structure of **MON-A** includes Si, C, O, and N, confirming the success of the cocondensation reaction between TEOS and urea-amine compound **1**.

**Figure 5:**
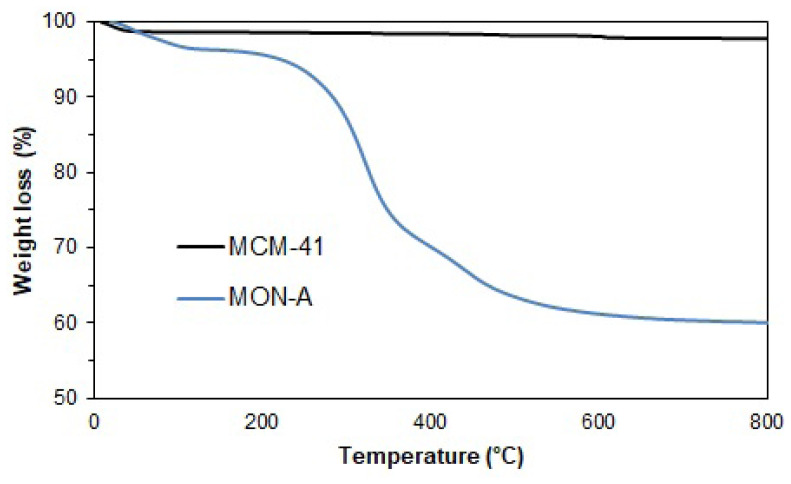
TGA curves of **MCM-41** and **MON-A**.

Figure 5 shows the TGA curve of **MCM-41**. The ~1% mass loss observed in the TGA curve of **MCM-41** below 200 °C is due to the physically attached solvents trapped in the pores in the channels of **MCM-41** and is consistent with the literature [35–37]. The ~0.8% mass loss observed between 200 and 800 °C may have occurred due to the dehydration of the free silanol groups on the surface of **MCM-41** at this temperature range [38,39]. Thermogravimetric analysis was also performed for **MON-A** in order to calculate the amount of organic part in its structure. The TGA curve of **MON-A** is shown in Figure 5 as well. The approximately 4% mass loss observed below 200 °C in the TG curve for **MON-A** is thought to have been due to solvents trapped and physically attached to the pores in the channels of the mesoporous material, as in the TG curve of **MCM-41**. The weight loss observed between 200 and 800 °C is thought to have been due to the degradation of the organic **1** structure attached by covalent bonds as a result of the cocondensation reaction to the silica network of **MON-A**. The observed mass loss in this region range was approximately 36%.

**Figure 6:**
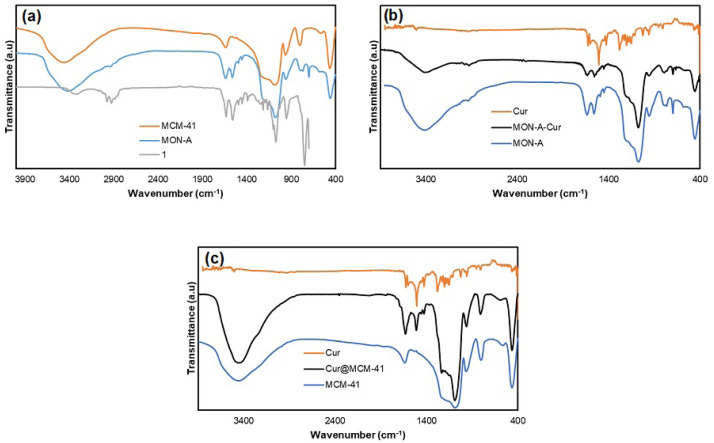
FT-IR spectra of a) **1**, **MCM-41** and **MON-A** and b) curcumin, **MON-A** and **MON-A-Cur** c) curcumin, **MCM-41** and **Cur@MCM-41**.

Finally, FTIR spectroscopy was used to characterize **MCM-41** and **MON-A** nanoparticles. The overlaid FTIR spectra of **MCM-41**, **MON-A**, and compound **1** are given in Figure 6a. In the FTIR spectrum of **MCM-41**, the asymmetric and symmetric stretching vibrations of the Si–O–Si bond were observed at 1105 and 801 cm^−1^, respectively, while the bending vibration was observed at 455 cm^−1^ [18]. The presence of silanol groups in this structure was characterized by the vibration band at 962 cm^−1^ [18]. Vibration bands for –NH_2_, C=O, CH_2_, and the aromatic ring were observed at 1553 cm^−1^, 1629 cm^−1^, 2915 cm^−1^, and 3053 cm^−1^ in the FTIR spectrum of compound **1** and these values are consistent with those given in the literature [12]. Compared with the FTIR spectrum of **MCM-41**, new vibrational bands were observed at 1564 cm^−1^ for the NH_2_ group, at 1635 cm^−1^ for the C=O group, at 2862 and 2928 cm^−1^ for the CH_2_ groups, and at 3025 cm^−1^ for the aromatic ring in the FTIR spectrum of **MON-A**. The observation of the same vibrational bands with small shifts in wavenumbers corresponding to the groups in the spectrum of compound **1** in the FTIR spectrum of **MON-A** suggested the successful synthesis of **MON-A**.

### 3.2. Curcumin loading into MON-A and MCM-41

Curcumin-loaded **MON-A-Cur** and **Cur@MCM-41** were obtained by mixing 25 mL of stock curcumin solution with 100 mg of **MON-A** and **MCM-41** at room temperature and in darkness for 24 h after the necessary washing and drying processes. Then the absorbances of the curcumin loading and washing solutions were read in the UV-Vis spectrophotometer at λ = 420 nm, and the amount of curcumin remaining in the solution was calculated using the standard calibration curve. Finally, the encapsulation efficiency for **MON-A-Cur** and **Cur@MCM-41** was calculated as 25% and 14% with the help of equation (1), respectively.

The FTIR spectra of bare mesoporous materials and curcumin-loaded mesoporous materials were recorded to see the changes in the FTIR spectrum after curcumin loading on nanocarriers **MON-A** and **MCM-41**. The FTIR spectra of curcumin, **MON-A**, and **MON-A-Cur** are given in Figure 6b, while the FTIR spectra of curcumin, **MCM-41**, and **Cur@MCM-41** are given in Figure 6c. The FTIR spectrum of free curcumin showed a number of fundamental vibrations related to the functional groups of curcumin such as OH at 3502 cm^−1^, C–H of the aromatic ring at 3030 cm^−1^, aliphatic C–H at 2954 cm^−1^, C=O at 1625 cm^−1^, aromatic C=C at 1602 cm^−1^, and C–O functional groups at 1284 cm^−1^. The FTIR spectra of **MON-A-Cur** and **Cur@MCM-41** were recorded as spectra with vibration signals of both curcumin and bare mesoporous materials together. This confirms that curcumin was successfully loaded into the **MON-A** and **MCM-41** structures.

### 3.3. In vitro curcumin release of MON-A-Cur and Cur@MCM-41 at different pH values

**Figure 7:**
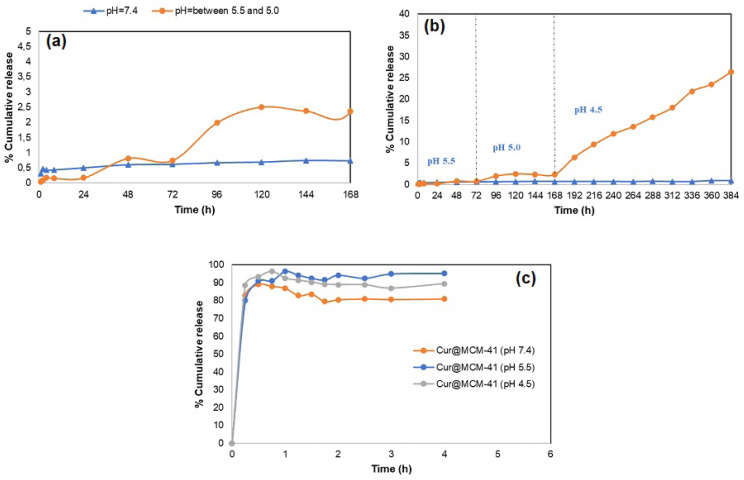
Curcumin release profiles from **MON-A-Cur** (a and b) and **Cur@MCM-41** (c) at different pH values.

To examine the pH effect on drug release from **MON-A-Cur**, an in vitro release study was performed at pH 7.4 and pH 5.5 For this, **MON-A-Cur** was dispersed in 10 mL of PBS (pH 7.4 and 5) containing 10% Tween-80 (v/v) and mixed at 37 °C at 100 rpm. Readings at λ = 420 nm were obtained in the UV-Vis spectrophotometer by taking samples from the drug release media at different times intervals. The read absorption value was plotted on the standard calibration curve and the percent release rates obtained were plotted against time. The same procedures were then repeated for **Cur@MCM-41**. Graphs summarizing the results obtained are given in Figure 7.

The release of curcumin from **MON-A-Cur** was recorded as only 0.5% at pH 7.4 after 168 h. In the experiments carried out at pH 5.5 for **MON-A-Cur**, a release value of 0.5% was obtained after 72 h (Figures 7a and 7b). The fact that curcumin release is 0.5% at physiological and endosomal pH indicates that the interactions between the nanocarrier **MON-A** and curcumin are strong enough to prevent curcumin leakage at these pHs. When the release experiments were repeated for **Cur@MCM-41** under the same conditions, the release of curcumin from **Cur@MCM-41** was 95% at pH 7.4 and more than 80% at pH 5.5 within the first 15 min (Figure 7c). The almost complete lack of release of curcumin from **MON-A** compared to **MCM-41** is attributable to possible strong interactions between **MON-A** and curcumin.

**Figure 8:**
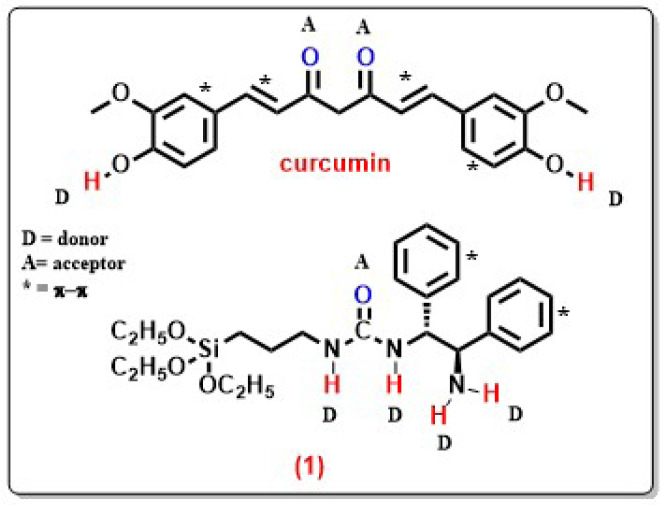
The possible interaction types between curcumin and **MON-A**.

The urea-amine **1** structure was used to obtain the mesoporous material **MON-A**. Figure 8 shows possible types of interactions between curcumin and compound **1**. In addition to the potential to form a large number of H bonds due to H donors and acceptors on both structures, π–π interactions may also occur due to the π-conjugated groups that both groups have. In addition to these noncovalent interactions, covalent bonding is also possible between the C=O and NH_2_ groups in curcumin and compound **1**, respectively, via a C=N bond with a Schiff base reaction. Curcumin release from the **MON-A-Cur** structure may not have occurred since pH 5.5 could not break down these noncovalent and covalent interactions. To test this, the pH of the drug release medium was adjusted to 5.0 after 72 h. In the measurements made during the subsequent 96 h, curcumin release from **MON-A-Cur** reached 2.5%. A decrease in pH from 5.5 to 5.0 resulted in a 5-fold increase in drug release. In order to determine whether the release of curcumin would start again with pH lowering, the pH of the release medium was lowered to 4.5 and aliquots were taken from drug release medium at certain time intervals for the next 216 h. The absorption values of the aliquots showed that curcumin release from **MON-A-Cur** was 26.3% after 216 h at pH 4.5. At the end of this period, the curcumin release still did not reach equilibrium, which indicates that it may take a long time for the curcumin release to reach equilibrium. The continuous and linear increase in curcumin release from **MON-A-Cur** at pH 4.5 indicates the presence of a covalent bond between curcumin and the **MON-A** carrier system via C=N bonds, because it is known that C=N bonds, which are characteristic in the formation of Schiff bases, are sensitive to pH changes and cleavable at acidic pHs [16,40]. The fact that lower pH values result in a higher release percentage indicates that the release of curcumin from **MON-A-Cur** is pH controlled. To see if the controlled release profile observed for **MON-A-Cur** at pH 4.5 could also be obtained for **Cur@MCM-41**, the curcumin release experiment for **Cur@MCM-41** was repeated under the same conditions. Figure 7c shows that more than 90% of curcumin was released from **Cur@MCM-41** in the first 15 min at pH 4.5. This result indicates that if the silica surface is functionalized in accordance with the drug to be transported, it creates a stronger interaction with the drug and allows the drug to be transported effectively.

## 4. Conclusion

We successfully synthesized and examined the **MON-A** drug delivery system functionalized with urea-amine groups, which does not leak curcumin at physiological and endosomal pH without pore-closing agents and performing pH-controlled and prolonged release of curcumin. In vitro release experiments performed with the **MON-A** system at different pHs showed that the imine bond formed after the Schiff base reaction between the carrier system and curcumin provides strong pH sensitivity to **MON-A**. The functionalization of mesostructured silicas with organic groups such as urea-amine makes significant contributions to loading hydrophobic drugs such as curcumin into these structures with higher capacity and providing the triggers required for the controlled release of drugs from these structures. The results obtained in our study showed that **MON-A** can transport curcumin without leakage and achieve controlled release of curcumin with pH sensitivity. Further studies on in vitro targeted release and other related studies are ongoing. However, many detailed studies on in vitro targeted drug release and cell culture are required to determine whether newly synthesized delivery systems such as **MON-A** are suitable for use in clinical trials.

## Figures and Tables

**Table:** The structural parameters of **MCM-41** and **MON-A**.

Material	*S*_BET_ (m^2^/g)^a^	*D_BJH_* (nm)^b^	*V_total_* (cm^3^/g)^c^
**MCM-41**	1147	2.7	0.61
**MON-A**	415	2.3	0.23

[a] *S*_BET_ = surface area, calculated by BET method.

[b] *D_BJH_* = pore diameter, calculated by BJH method.

[c] *V_total_* = pore volume.
